# Foul Language in Public Places

**Published:** 1892-12-31

**Authors:** 


### PASSING TOPICS.

### FOUL LANGUAGE IN PUBLIC PLACES.

In the present state of public opinion, the question, how to abate the terrible nuisance caused by the use of foul language in the streets may well be accounted a burning one. It is difficult to write of this plague with strict sobriety, and we may safely assert that under an infliction of this character the respectable members of a civilised community are placed at a positive disadvantage as compared with the inhabitants of a fresher and purer, if rougher, land, where the soil is not yet saturated with the moral scourge of centuries, and the winds blow sweetly from the Delectable mountains without passing over leagues of contaminated area.

It is but a few weeks ago that we commented upon the increasingly disgraceful behaviour of children attending Board and other day schools. We are not inclined to treat the subject with cynicism, and yet it is difficult to separate the lofty pretensions of this prodigiously educational age from its actual achievements in conduct and morality. A system of national teaching has been in existence now for a sufficient number of years to bear fruit. And, without incurring a charge of undue haste, the nation may reasonably inquire what is the quality of the grain which has been produced at such vast expenditure and by the aid of so complex machinery? Well, we have only to walk in our streets and to keep our eyes and ears open in order to sample it for ourselves. We cannot put the case more strongly than by echoing the humiliating complaints of indignant correspondents, that no decent person can make use of our suburban thoroughfares—to say nothing of certain of the great metropolitan roadways, or the streets of our villages—without being revolted by foul and blasphemous language.

If it be the chief aim and object of national education to produce good citizens, we are forced to conclude that failure has attended the efforts put forward in this direction, because the very saddest feature of the situation is that the most obtrusively obnoxious section of the population is the young—those who have been the objects of our legislators' solicitude, and who were supposed to have started life endowed with benefits for lack of which their parents had been condemned to languish. Yet the fathers and mothers who grew up without the more ample instruction vouchsafed to their children had at least this advantage—they were taught to fear God and to honour the King. In the foolish attempt to satisfy schismatics, atheists, and sedition mongers, we have banished religious training, and with it moral restraint, and unless the respectable members of all classes are to be swallowed in the black tide rapidly rising around them, they must cease to be content with stopping their ears and affecting not to notice that which is only too grossly evident. They must be prepared to retaliate. The law as it stands is, we believe, sufficient if its action be invoked; if not, let our rulers be made aware that the nation demands some remedy for social evils which are poisoning the lives of the people, while the time and opportunities of its legislators are too often expended upon what by comparison appear to be contemptible party issues.

